# Progress in the Development of Subunit Vaccines against Malaria

**DOI:** 10.3390/vaccines8030373

**Published:** 2020-07-10

**Authors:** Mariusz Skwarczynski, Saranya Chandrudu, Berta Rigau-Planella, Md. Tanjir Islam, Yee S. Cheong, Genan Liu, Xiumin Wang, Istvan Toth, Waleed M. Hussein

**Affiliations:** 1 School of Chemistry and Molecular Biosciences, The University of Queensland, St Lucia, QLD 4072, Australia; m.skwarczynski@uq.edu.au (M.S.); saranya.chandrudu@uqconnect.edu.au (S.C.); berta.rigau7@gmail.com (B.R.-P.); mdtanjir.islam@uq.net.au (M.T.I.); yee.cheong@uq.net.au (Y.S.C.); genan.liu@uq.net.au (G.L.); wangxiumin@caas.cn (X.W.); 2 Gene Engineering Laboratory, Feed Research Institute, Chinese Academy of Agricultural Sciences, Beijing 100081, China; 3 Key Laboratory of Feed Biotechnology, Ministry of Agriculture and Rural Affairs, Beijing 100081, China; 4 School of Pharmacy, Pharmacy Australia Centre of Excellence, The University of Queensland, Woolloongabba, QLD 4072, Australia; 5 Institute for Molecular Bioscience, The University of Queensland, St. Lucia, QLD 4072, Australia

**Keywords:** malaria, parasite, *Plasmodium*, peptide-based vaccine, clinical trials

## Abstract

Malaria is a life-threatening disease and one of the main causes of morbidity and mortality in the human population. The disease also results in a major socio-economic burden. The rapid spread of malaria epidemics in developing countries is exacerbated by the rise in drug-resistant parasites and insecticide-resistant mosquitoes. At present, malaria research is focused mainly on the development of drugs with increased therapeutic effects against *Plasmodium* parasites. However, a vaccine against the disease is preferable over treatment to achieve long-term control. Trials to develop a safe and effective immunization protocol for the control of malaria have been occurring for decades, and continue on today; still, no effective vaccines are available on the market. Recently, peptide-based vaccines have become an attractive alternative approach. These vaccines utilize short protein fragments to induce immune responses against malaria parasites. Peptide-based vaccines are safer than traditional vaccines, relatively inexpensive to produce, and can be composed of multiple T- and B-cell epitopes integrated into one antigenic formulation. Various combinations, based on antigen choice, peptide epitope modification and delivery mechanism, have resulted in numerous potential malaria vaccines candidates; these are presently being studied in both preclinical and clinical trials. This review describes the current landscape of peptide-based vaccines, and addresses obstacles and opportunities in the production of malaria vaccines.

## 1. Introduction

Malaria is a major health problem worldwide and is one of the most prevalent diseases in developing countries. Globally, it is responsible for around half a million deaths annually. People living in high-poverty areas, where rainfall and temperature favor the transmission of *Plasmodium* parasites, are at the highest risk of contracting malaria and developing severe disease symptoms [1,2]. Human malaria is commonly caused by four different *Plasmodium* species: *P. falciparum*, *P. vivax*, *P. malariae*, and *P. ovale*. Severe cases of malaria in sub-Saharan Africa are typically caused by *P. falciparum*, while infections in southeast Asia almost always result from *P. vivax* and *P. falciparum* [3,4,5,6]. Young children and infants are especially susceptible to malaria due to their lack of acquired immunity. Risk is also greater in pregnant women, as pregnancy represses the immune system [7].

Two major prophylactic strategies are used to control malaria: avoiding bites by mosquitoes that carry parasites (prevention of the infection), and therapeutic use of antimalarial drugs (prevention of the disease). Mosquito nets can prevent insects from reaching a host, but this approach is far from sufficient in terms of eradicating malaria. It was estimated that less than 2% of African children were saved by insecticide-treated nets (ITNs) [8]. Other control measures, such as spraying insecticides (dichlorodiphenyltrichloroethane, DDT, pyrethroids, permethrin and deltamethrin) on the walls of homes as recommended by the World Health Organization (WHO), have also been used. However, it is impossible to completely eradicate mosquitoes. During the global malaria eradication program launched by WHO in 1955 [9], chloroquine was used for prevention and treatment, while DDT was used for mosquito control. Both were utilized on a massive scale. This significantly reduced malaria mortality in certain countries, such as India and Sri Lanka [10]. However, the program was abandoned in 1972 due to the emergence of *Plasmodium* parasites resistant to chloroquine and *Anopheles* mosquitos resistant to DDT [11,12].

Several other chemical drugs (e.g., mefloquine, halofantrine) and herbal compounds (e.g., artemisinin and its derivatives) are currently being used to treat malaria; however, *Plasmodium* parasites are becoming resistant to these agents as well [13,14]. Therefore, artemisinin-based combination therapy (artemisinin with lumefantrine, mefloquine, amodiaquine, sulfadoxine/pyrimethamine, piperaquine and chlorproguanil/dapsone) has recently been recommended by WHO for infections caused by *P. falciparum*.

Antibiotics have also been extensively studied as a treatment option for malaria. Doxycycline, clindamycin, and azithromycin have shown to be effective against malaria and reached clinical application; however, they are all delayed action drugs and are, therefore, not effective in delivering the immediate therapy required for advanced malaria cases. Several other antibiotics have been also investigated, including co-trimoxazole, fusidic acid, erythromycin, tetracycline, tigecycline, mirincamycin, ciprofloxacin, quinolones, ketolide, thiopeptides, nocathiacin, sulfamethoxazole, trimethoprim, tigecycline, and telithromycin. These have been effective in killing malaria parasites; however, most of them are still in the early stages of development [15,16].

Despite all of the efforts to control malaria at both the human and mosquito levels, an estimated 228 million cases of malaria were contracted in 2018, with 405,000 deaths worldwide. Contemporary malaria cases are notably exacerbated by the emergence of insecticide-resistant mosquitos and drug-resistant parasites [17]. Furthermore, even when treatment could be effective and life-saving, the new generation of drugs needed are often too expensive for people in developing countries to afford. 

In addition to drug-based disease control, the human immune system (including innate, humoral, and cellular immunity) can be exploited for the prevention of malaria. Following repeated exposure to malaria-causing parasites, people (usually those living in malaria-endemic zones) can eventually develop naturally acquired immunity [18,19]. These immune responses build gradually and are influenced by a variety of factors, such as the age of the host, epidemiology, and parasite species/stage. However, natural immunity does not provide long-term protection against malaria [20,21].

**Figure 1:**
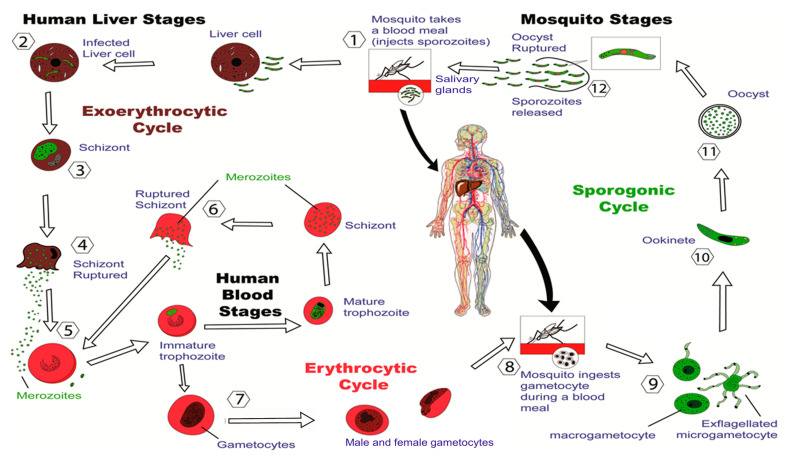
Life cycle of *Plasmodium* malaria parasites. Malaria parasites have a complex, multi-staged life cycle that occurs in two living beings: the vector mosquitoes and vertebrate hosts. (1) *Plasmodium* parasites enter the blood of their vertebrate host when an infected female *Anopheles* mosquito bites a person and injects the parasites in the form of sporozoites. (2) The sporozoites enter into the bloodstream and head immediately for the liver, where they invade liver cells. (3–4) The parasites reproduce in the liver to produce thousands of haploid forms, called merozoites. (5) The merozoites emerge from the liver and re-enter the bloodstream, where they infect red blood cells (RBCs). (6) They continue to reproduce asexually in the RBCs and release newly formed merozoites back into the bloodstream. Thousands of parasite-infected cells in the host’s bloodstream can lead to symptoms, such as recurrent fever, chills, headache, anemia, fatigue, perspiration, anorexia, and vomiting. (7) Some merozoites develop into male and female gametocytes, which are the sexual forms of the parasite. (8–9) When a mosquito has a blood meal from an infected person, these gametocytes are ingested in the gut of the mosquito. (10) Male and female gametes fuse together to form a diploid zygote, which develops into ookinetes that burrow in the midgut wall of the mosquito to form oocysts. (11–12) After a few days, oocysts burst and release sporozoites into the body cavity of the mosquito. These sporozoites then invade the salivary glands of the mosquito. The cycle continues when the mosquito bites an appropriate host, injecting the sporozoites into the animal’s bloodstream [26,27,28].

The gravity of the statistics around malaria has prompted and justified intensive research into developing a safe, widely-affordable and effective vaccine against malaria. Currently, there are three major approaches for the development of malaria vaccines that correspond to the three stages of the parasite’s life cycle (Figure 1), namely (a) pre-erythrocytic, (b) erythrocytic, and (c) mosquito vector (blocking transmission of the parasite). Pre-erythrocytic-stage vaccines predominantly aim to protect the host against initial infection. These vaccines target the liver stage of the parasites: sporozoites that were introduced by the mosquito. Pre-erythrocytic vaccines often include radiation-attenuated sporozoites. They have been able to induce some protective immunity against malaria in rodents [22,23] and humans [24,25]. Erythrocytic, or blood-stage vaccines, aim to reduce morbidity and mortality associated with the disease when parasites are present in red blood cells. Transmission-blocking vaccines aim to block malaria transmission from mosquitoes to humans by preventing the malaria parasite from developing in the mosquito.

Only one commercial antimalarial vaccine presently exists, RTS,S/AS01. It was approved in 2015; however, it has low efficacy (26–50%) [29]. In addition, human responses to the vaccine, both in the field and in controlled human malaria infections in non-endemic settings, are extremely variable. RTS,S includes a *P. falciparum* circumsporozoite (CS) protein carboxy-terminal fragment bonded to the surface antigen of hepatitis B (HBsAg). These antigens are assembled into particulate structures. The RTS,S was developed using the adjuvant AS01, which includes monophosphoryl lipid A (MPL) and saponins mixture QS21 in liposome-based formulation. RTS,S/AS01 was assessed in a phase III trial [30] and while it received a positive recommendation from the European Medicines Agency, further improvements into the vaccine’s properties were recommended due to its limited efficacy and the fact that the fundamental mechanism of action is still not well-understood [31].

In order to direct further development and advancement in malaria vaccine strategies, it is important to consider which factors may lead to the variation in efficacy in humans. Nielsen et al. analyzed the data from six phase II trials of RTS,S [32], in which the correlation between human leukocyte antigen (HLA) allele groups and the protection mediated by RTS,S was evaluated using linear regression and multivariate logistic. Significant associations were found between the HLA-A/01, HLA-B/08 and HLA-DRB1/15/16 allele groups and positive protection results; whereas other allele groups, such as HLA-A/01, HLA-B/53 and HLA-DRB1/07, were associated with insufficient protection. It is worth noting that these “protective” allele groups are known to have a lower prevalence in sub-Saharan African populations compared to populations in United Kingdom or United States, where these phase II RTS,S trials took place. This finding illustrated how the HLA genotype may affect the protective efficacy of RTS,S against malaria infection [32].

Overall, the difficulty in developing effective malaria vaccines stems largely from the complexity of the malaria-causing parasites’ life cycle, which includes mosquitos, human liver, and human blood stages (Figure 1), and subsequent antigenic variations of the parasite [33]. These parasites are also able to hide inside human cells to avoid being recognized by the immune system, creating further challenges [34].

Conventional vaccines use either live-attenuated/killed pathogens or recombinant protein to evoke an immune response [35,36]. The disadvantages of these vaccines include possible problems in protein expression, contamination from the expression process, difficulty in pathogen cultivation (which is especially problematic for malaria), and the risk of autoimmune and excessive inflammatory responses in humans. Therefore, peptide-based vaccine approaches have become very attractive due to: (i) the relative ease in producing peptides in large-scale with high purity; (ii) the ability for these vaccines to be freeze-dried and stored in solid form at room temperature; (iii) the possibility to target appropriate immune responses through vaccine customization; and (iv) greatly reduced risk of autoimmune and allergic responses [37]. Peptide-based vaccines are generally designed based on the minimal peptide epitope that is still able to trigger the desired immunity. Peptide-based vaccines can suffer the disadvantage of being sensitive to any mutation within the selected epitopes and too genetically constrained due to the specificity of the T-cell receptor (TCR) [38]. Further, low immunogenicity is a typical weakness of peptide-based vaccines; however, it can be minimized through the development of better adjuvant-based delivery systems.

**Figure 2:**
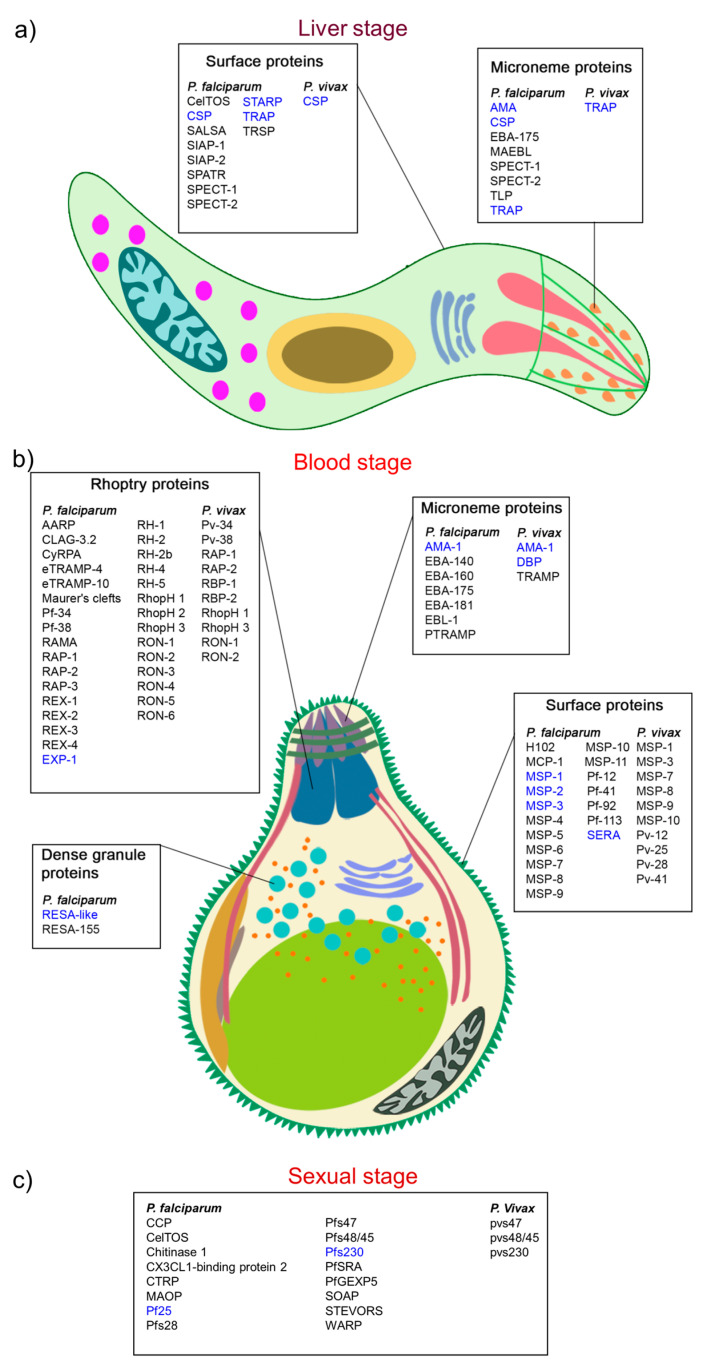
Potential antigenic proteins of *Plasmodium*. (**a**) The location of potential preerythrocytic liver-stage antigenic *Plasmodium* sporozoite proteins in *P. falciparum* and *P. vivax*. Surface proteins: SALSA: sporozoite and liver-stage antigen; SIAP: sporozoite invasion-associated protein; SPATR: secreted protein with altered thrombospondin repeat; SPECT: sporozoite proteins essential for cell traversal; STARP: sporozoite threonine and asparagine-rich protein; TRSP: thrombospondin-related sporozoite protein; Microneme proteins: AMA: apical membrane antigen; CSP: circumsporozoite protein; TLP: TRAP-like protein; TRAP: thrombospondin-related anonymous protein. (**b**) The location of potential erythrocytic-stage malaria antigenic *Plasmodium* merozoite proteins in *P. falciparum* and *P. vivax* [41,42,43,44]. Rhoptry proteins: AARP: apical asparagine-rich protein; CLAG: cytoadherence-linked asexual gene protein; CyRPA: cysteine-rich protective antigen; eTRAMP: early transcribed membrane protein; EXP: exported protein; Pf: *Plasmodium falciparum* antigen; RAMA: rhoptry-associated membrane antigen; RAP: rhoptry-associated protein; RBP: reticulocyte-binding protein; REX: ring-exported protein; RhopH: high-molecular mass rhoptry protein complex; RON: rhoptry neck protein; RH: reticulocyte binding-like homologue protein; Microneme proteins: DBP: Duffy-binding protein; EBA: erythrocyte-binding antigen; EBL: erythrocyte-binding ligands; PTRAMP: *Plasmodium* thrombospondin-related apical merozoite protein; TRAMP: thrombospondin-related apical merozoite protein. Dense granule proteins: RESA: ring-infected erythrocyte surface antigen; Surface proteins: MCP: merozoite capping protein; MSP: merozoite surface protein; Pv: *P. vivax* antigen; SERA: serine repeat antigen. (**c**) The location of potential sexual-stage malaria antigenic *Plasmodium* merozoite proteins in *P. falciparum* and *P. vivax* [45,46]. CCP: Plasmodium falciparum LCCL domain-containing protein; CelTOS: Cell-traversal protein for ookinetes and sporozoites; CTRP: Circumsporozoite and thrombospondin-related anonymous protein; MAOP: Membrane-attack ookinete protein; PfSRA: Plasmodium falciparum surface related antigen; PfGEXP5: Plasmodium falciparum Gametocyte Exported Protein-5; SOAP: Secreted ookinete adhesive protein; WARP: Von Willebrand factor-A domain-related protein.

The first parenteral peptide-based malaria vaccine, which was tested clinically in 1987, was designed based on repeat peptide sequences (NANP)3 derived from *P. falciparum* circumsporozoite protein (CSP) [39]. Since then, a variety of synthetic peptide vaccines have been developed for both murine (*P*. *berghei* and *P*. *yoelii*) and human (*P*. *falciparum* and *P*. *vivax*) parasites and tested for immunogenicity and efficacy. Several of these reached advanced clinical trials [40]. Additionally, the development of a database of nearly 561,924 unique B-cell and T-cell epitopes from *Plasmodium* proteins (Figure 2), including those from human *P*. *falciparum* and *P*. *vivax* parasites, has provided researchers with a comprehensive repository of potential peptide targets (http://www.immuneepitope.org). 

This review outlines major antigenic targets for malaria peptide-based vaccine design and discusses the challenges and prospects of vaccine development.

## 2. Peptide-Based Vaccines

### 2.1. Pre-Erythrocytic-Stage Vaccines

The pre-erythrocytic parasite stage is the most advantageous target for vaccine development against malaria because the inhibition of the liver stage of parasite growth blocks merozoites from entering the bloodstream. As such, symptomatic malaria can be prevented (Table 1).

#### 2.1.1. Circumsporozoite Protein

Studies in transgenic mice that were tolerant to CSP T-cell epitopes confirmed that CSP is the immunodominant target of protective immune responses elicited by irradiated sporozoites [63]. Neutralizing antibodies against sporozoites targeted the species-specific central repeat region of CSP, and the antibodies recognizing the anti-repeat region bound to the surface of the sporozoites, inhibited motility, and blocked invasion of the host’s hepatocytes [64].

RTS,S (licensed by GlaxoSmithKline Biologicals) is the only approved malaria vaccine on the market. It is based on the CSP of *P. falciparum* 3D7 clone. The vaccine antigen is comprised of two peptides; RTS and S, which are recombinantly co-expressed in *Saccharomyces cerevisiae* (Table 1) [48,65]. RTS corresponds to amino acids 207–395 of CSP. It is conjugated to S, which is the N-terminus peptide of HBsAg, which consists of 226 amino acids. The RTS,S vaccine specifically targets the pre-erythrocytic stage of *P. falciparum*. It confers a moderate to low level of protection against infection by *P. falciparum* sporozoites in humans [47,66].

An improved version of this vaccine, RTS,S/AS02, was developed based on a new adjuvant (AS02, an oil in water-based formulation containing the immunostimulants MPL, a nontoxic derivative of lipopolysaccharide (LPS), and QS21). RTS,S/AS02 achieved elevated antibody titers and enhanced cell-mediated immune responses in both malaria-naive and malaria-experienced individuals [48]. A separate phase III clinical trial of RTS,S/AS01 (NCT00866619) was conducted in Africa with 6537 infants (age 6–12 weeks) and 8923 children (age 5–17 months). In this study, parasite clearance occurred in 46% of vaccinated children and 27% of infants [30]. Therefore, further improvements are needed to potentiate the efficacy of RTS,S. Although RTS,S/AS01 has been approved for active immunization against malaria in children between the ages of 6 weeks to 17 months by the European Medicines Agency, WHO has not recommended the implementation of RTS,S/AS01 in the Expanded Program on Immunization (EPI) [29].

Alternative versions of RTS,S equivalent vaccines have also been examined for *P. vivax* circumsporozoite (CSV) protein and a truncated region containing repeat sequences from both VK210 and VK247 subtypes of the parasite expressed in *E. coli* [60]. CSV-S,S included CSV-S, a fusion protein between VMP001 and S (HBsAg peptide) expressed in *S. cerevisiae*. CSV-S,S/AS01 induced higher levels of vaccine-specific antibodies than VMP001/AS01 in rhesus monkeys [60]. However, no challenge study was performed.

Parra-López et al. designed a highly conserved HLA-DRβ1*04:01 (DR4) epitope (QNT-5_332–345_) situated at the C-terminus of a CD4 T-cell epitope named T* (*P. falciparum* CS_326–345_). HLA-DR4 transgenic mice were immunized with QNT-5_332–345_ [49] to elicit long-term anti-CS antibody responses and prime CD4^+^ T-cells in mice. To improve QNT-5_332–345_ immunogenicity, the P1 anchor position of the epitope was substituted with a tyrosine residue and named QNT-Y. QNT-Y peptide interacted strongly with major histocompatibility complex II (MHCII) receptors and formed stable MHC-peptide complexes. Disappointingly, IFN-γ and antibody responses induced by linear QNT-Y-containing peptide were significantly lower than those of wild-type QNT-5 peptide [49]. 

Microparticle peptide-based malaria vaccines were developed by Powell et al. using layer-by-layer (LBL) fabrication of polypeptide films on solid CaCO_3_ cores [50]. These vaccines were comprised of tri-epitope CS peptide T1BT*, including the B-cell epitope of the CS repeat region B and the highly conserved T-cell epitope (T1), as well as the universal epitope T* that is detected by multiple molecules of HLA class II [67]. Parasite-neutralizing antibodies and malaria-specific T-cell responses, including cytotoxic effector T-cells, were produced upon immunization of mice. When the challenge was performed with live sporozoites from infected mosquitoes, protection from infection was found to be associated with levels of neutralizing antibodies. However, some immunized mice produced low or undetectable neutralizing antibodies levels, yet were still protected. Furthermore, mice immunized with only T-cell epitopes carrying microparticles developed parasitemia, indicating that cellular immunity alone is not sufficient for preventing infection. The immune response against this vaccine was potentiated with the incorporation of the TLR2 agonist Pam_3_Cys [50,68,69].

Mitchell et al. used the skin scarification technique to deliver malaria repeat peptide containing a protective B-cell epitope of *P. falciparum* CS formulated with TLR-7/8 and -9 agonists [51]. The vaccine elicited high levels of neutralizing antibodies, triggered protective cell-mediated immunity in mice, and resulted in mice resistant to disease when challenged through bites by infected mosquitoes. In this study transgenic *P. berghei* sporozoites expressing *P. falciparum* CS repeats were used for infection of mosquitoes.

Wilson et al. evaluated Poly I:C and Montanide as adjuvants and polystyrene nanoparticles as a carrier for the delivery of the immunodominant CD8 T-cell epitope, KI (SYIPSAEKI), of *P. berghei* CSP (named pb9 or KI) in the murine model. The systems induced the production of IFN-γ and cellular immunity [70]. The highest CD8 T-cell responses were induced against KI vaccine adjuvanted with Montanide or covalently conjugated to polystyrene nanoparticles (40–50 nm), rather than adjuvanted with Poly I:C or delivered as a mixture with polystyrene nanoparticles.

Several other epitopes derived from CS protein were examined; however, immune responses were poor, non-selective, or the vaccines were not protective [71,72,73].

#### 2.1.2. Other Liver-Stage Peptide Specific Sequences

Liver-stage antigen 1 (LSA-1) is expressed during the liver stage of malaria in the parasitophorous vacuole [74]. It consists of 17 amino acid repeat units (ALKEKLQ-X-QQSDLEEQR, wherein X is Glu or Gly) and plays a vital role in late liver-stage schizogony [74,75,76]. LSA-1 naturally evokes antibody responses, especially those of the IgG1 subclass, in humans exposed to *P. falciparum* infection [57]. Recombinant LSA-1, either alone or in combination with AS01/AS02, elicited high antibody titers in humans and induced CD4^+^ cells to produce IFN-γ and IL-2, but did not protect against *P. falciparum* infection [77]. However, liver-stage antigen 3 (LSA-3) was identified following immunization with irradiated sporozoites in humans [42,78]. Immunization with LSA-3 in chimpanzees induced full protection against successive heterologous challenges with a large numbers of *P. falciparum* sporozoites [58]. Efficacy of the vaccine candidate has not yet been demonstrated in humans [79].

### 2.2. Erythrocytic-Stage Vaccines

Malaria parasites attack red blood cells (RBCs) during their merozoite stage. RBCs cannot stimulate T-cell responses, as they do not express MHC class I molecules. Thus, parasite infection at this stage can generate only humoral immune responses. Two types of antibodies are produced against merozoites at the blood stage: (a) antibodies that recognize antigens from merozoite surface proteins and (b) antibodies that recognize parasite antigen expressed on infected RBCs, which initiates Antibody-Dependent Cell-Mediated Cytotoxicity [80]. The blood stage vaccines are designed based on these antigens (Table 2); among which MSP-1, MSP-2 and MSP-3 are the most commonly employed [81].

#### 2.2.1. MSP and Glutamate-Rich Protein

MSP-1 is expressed from the schizogony stage of malaria parasites and is involved in RBC invasion by parasites in the merozoite stage [88]. MSP-1 has highly conserved regions recognized by B- and T-cells in both human and murine models. To identify potent vaccine candidates, two synthesized multiple epitope peptides (MEP), P1 and P2, containing B- and T-cell epitopes from two N-terminal conserved regions of blood-stage antigen MSP1 and RESA (ring-infected erythrocyte surface antigen) of *P. falciparum*, respectively, were tested in mice [89]. Both P1 and P2 were able to stimulate high levels of antibody titers without the need for any carrier proteins. Upon challenge with a lethal dose of *P. yoelii nigeriensis* blood-stage parasites, only P1 adjuvanted with alum provided protection in BALB/c mice. In contrast, P1 adjuvanted with theoretically much stronger adjuvant, complete Freund’s adjuvant (CFA), conveyed partial protection.

MSP-2 is a 45 to 52 kDa parasite integral membrane protein located on the merozoite surface. It has highly conserved N- and C-terminal regions that are recognized by human and murine immune cells. B-cell epitope (SNTFINNA) from the N-terminal conserved region of MSP-2 conjugated to the entire N-terminal peptide sequence (KNESKY-SNTFINNA-YNMSIRRSM), but not peptides separately, was found to be immunogenic in both BALB/c and C57BL/6 mice [90]. BALB/c mice immunized with the conjugate adjuvanted with CFA were able to resist challenge with *P. yoelii* 265BY strains, while C57BL/6 mice did not generate protective immunity.

Protective effects of human antibodies against MSP-3 and glutamate-rich protein (GLURP) have been suggested by a number of immuno-epidemiological studies. This followed demonstration that the levels of two antigen-specific cytophilic antibodies (IgGl and IgG3) were significantly associated with a reduced incidence of malaria [82,83,84]. The malaria vaccine candidate GMZ2 was designed as a hybrid polypeptide to include the N-terminal region of GLURP linked with the C-terminal region of MSP-3 [85]. GMZ2 adjuvanted with aluminum hydroxide induced high levels of IgG antibodies. Both malaria-naive adults and malaria-exposed preschool children produced vaccine-specific antibodies with broad inhibitory activity against geographically diverse *P. falciparum* isolates [85]. A recent phase II trial of GMZ2/aluminum hydroxide vaccine with a cohort of 1849 children demonstrated that GMZ2 was well-tolerated and induced high antibody titers, which unfortunately corresponded with poor protection against malaria (14%) [91,92,93].

#### 2.2.2. SPf66

SPf66 was one of the first synthetic peptide-based vaccines developed against malaria. It is a synthetic 45 amino acid peptide based on four *P. falciparum* proteins, including three asexual blood-stage protein fragments (83, 55 and 35 kDa), linked by the repeat region of circumsporozoite protein. The vaccine construct is primarily intended to target blood-stage malaria parasites. Initially, two studies were conducted to evaluate the efficacy of SP66 in humans [94,95]. The first study evaluated the immune response induced by SPf66 in humans from the Colombian Pacific coast. The Spf66 vaccine construct was able to induce higher antibody titers and protection against parasites after the third boost. The second study was conducted with 9957 children aged less than 1 year old to evaluate vaccine safety. Clinical observation was carried out 30 min and 48 h after each immunization and no adverse reactions were recorded in 97% of the cases. When tested in Colombian volunteers who were semi-immune, the vaccine demonstrated 38–60% protective efficacy against *P. falciparum* [96]. However, subsequent studies with participants from Tanzania [97], Gambia [98] and Thailand [99] found that Spf66 failed to provide protection against *P. falciparum* malaria. Following these results, justification could no longer be made for further trials of SPf66 using the same formulation [41,86].

#### 2.2.3. Apical Membrane Antigen (AMA-1)

AMA-1 from *P. falciparum* is one of the most promising target antigens for the development of erythrocytic-stage malaria vaccines (Figure 2) [53]. The entire region of this protein can be divided into three domains; the majority of antibodies recognize strain-specific epitopes in domain I. AMA-1-derived peptide comprising residues 446 to 490 was conjugated to the influenza virosome and tested in a murine model. All mice immunized with this virosome produced high antibody (IgG) levels, which were able to inhibit *P. falciparum* growth in vitro (95%).

In another study, virosomal formulations of peptide-phosphatidylethanolamine peptidomimetic conjugates, including the NANP repeat region of *P. falciparum* CSP (*Pf*CSP, UK-39) and *P. falciparum* AMA-1 (*Pf*AMA1, AMA49-C1), were administered to immunize healthy malaria-naive adults [55]. UK-39- and AMA49-C1-loaded virosomes induced a long-lasting parasite growth-inhibiting antibody response in humans. The antibodies from immunized volunteers inhibited sporozoite migration and the invasion of hepatocytes in vitro, suggesting the potential of influenza virosomes as a human-compatible antigen delivery platform for the development of subunit vaccines [54]. To investigate the efficacy of virosome-formulated *P. falciparum* AMA-A- and CSP-derived peptides as a malaria vaccine, ten Tanzanian adults and 40 children aged between 5 and 9 years old living in a malaria-endemic region were immunized with one or two doses of the vaccine on days 0 and 90. After vaccination, the incidence rate of clinical malaria episodes in children was reduced by 50% compared with control children. Only two adults showed malaria episodes. While it appears that this virosomal formulation has higher efficacy in adults, a larger-scale clinical study is required to test this observation.

## 3. Recent Clinical Trials, Challenges and Prospects

### 3.1. Clinical Trials

Over 20 malaria vaccines have been recently evaluated in clinical trials (Table 3). These were mostly designed to protect humans against *P. falciparum* infection. Only one, ChAd63/MVA PvDBP, was against *P. vivax*, and it has only reached phase I trials. More than dozens of malaria parasite proteins (or their fragments) have been selected as antigens. For example, SE36 is a malaria vaccine candidate bearing the N-terminal 47 kDa domain of *P. falciparum* serine repeat antigen 5 (*Pf*SERA5) [100]. SE36 inhibited parasite growth in vitro; however, there was a negative correlation between parasite density and antibody level [100,101]. In phase Ib trials, BK-SE36 vaccine induced 72% protective efficacy after 130–365 days post-second vaccination for Ugandan children aged 6–20 months who suffered from symptomatic malaria [102]. A leading alternative or potentially complementary strategy in fighting malaria is heterologous prime-boost immunization with sequential administration of chimpanzee adenovirus serotype 63 (ChAd63) and modified Ankara (MVA) vaccines. Both encode the malaria antigenic sequence called ME-TRAP. Venkatraman et al. conducted a controlled phase I study to evaluate the immunogenicity and safety of a ChAd63 vaccine candidate containing Matrix-M as an adjuvant [103,104]. Matrix-M is a promising Quillaja saponins-based vaccine adjuvant with demonstrated acceptable safety [105] and the ability to improve both cellular and humoral immune responses of vaccines [104,106,107]. In the abovementioned study, 22 healthy individuals were intramuscularly immunized with either (a) ChAd63-MVA ME-TRAP alone, or adjuvanted with (b) 25 µg or (c) 50 µg of Matrix-M. All groups received boost vaccination 8 weeks later. The group that received 50 µg of Matrix M had a significant increase in cytokine production at day 63. In all three groups, IgG production related to TRAP was highest 28 days after the boost vaccination. The vaccine was clinically safe and well-tolerated, with most local and systemic adverse reactions being mild in nature. Matrix-M did not increase local adverse effects; however, systemic adverse reactions were more frequently reported by volunteers receiving adjuvanted vaccine compared to the control group [103].

### 3.2. Challenges and Prospects

Both cellular and humoral immune pathways play significant roles in defending against malaria. It is important to note that scientists are still not able to articulate clear correlates of protection [108]. Therefore, the major impact of the various immune mechanisms remains to be defined, and as such all factors of the immune response are still under consideration in the design of vaccination strategies against this complex parasite disease [109].

Three decades following the first clinical trials on malaria vaccine, we are still yet to develop an effective strategy for prevention of the disease. However, a large number of peptide antigens have been identified. Pre-erythrocytic vaccines based on these peptides have delivered protection to some extent, or have at least led to reduced parasite growth [73]. Yet, research and development of malaria vaccines for liver and blood stages of the parasites face enormous challenges, as many vaccine candidates are poorly immunogenic and have shown toxicity in mice. *Plasmodium* can evade immune responses via a few reported mechanisms [110], therefore, non-natural (absent in infected individuals) immune responses stimulated by vaccine could overwhelm *Plasmodium* defense systems [111,112].

RTS,S is the first malaria vaccine able to develop significant immunity against malaria parasites. Early success with RTS,S led to reduced priority for, and less funding being directed at vaccines targeting blood-stage malaria, delaying vaccine development for infection by parasites at this stage. However, once RTS,S was found to have poor immunogenicity in women and children and increased pediatric morbidity, research interest in blood-stage malaria vaccines significantly intensified [30,113,114].

Another challenge faced in malaria vaccine development is that the common human malaria-causing parasite, *P. falciparum*, is not a rodent pathogen. The most common mouse models of malaria employ the rodent-specific parasite species *P. berghei*, *P. yoelii*, and *P. chabaudi*. These species generate unique pathologies and immune responses. While they are still employed to model various manifestations of human disease, the immune response patterns observed in these models are not fully transferable to humans [73].

For future efforts, many of the major problems in vaccine development can be minimized through the following: (a) using new adjuvants to improve immunogenicity; (b) discovery of new potent classical and cryptic epitopes [115] from a variety of *P. falciparum* proteins [116,117,118,119]; (c) modification to, and development of new, vaccine delivery systems, for example, nanoparticulate liposomal forms [112]; and (d) the use of multi-antigenic vaccines, including multiple epitopes from both blood and liver stages of malaria [57,119].

## 4. Conclusions

Malaria disease prevalence has a significant impact on society, resulting in high demand for vaccines against the disease. This is especially true for developing countries, where malaria is most detrimental. Low immunogenicity, adverse effects and storage limitations are some of the major challenges for malaria vaccine development. One of the most common targets for malaria vaccine development is sporozoite surface protein CSP. However, this might prove to be a “dead end” target, as a variety of vaccines containing CSP have failed to produce the desired protection.

Many vaccine candidates are currently in clinical trials. For example, ChAd63-MVA completed phase I clinical trials, showing high efficacy in the induction of cellular immunity against malaria with no evidence of dose-limiting toxicity. This vaccine is currently in phase II clinical trials. Peptide vaccines, especially those bearing strong immune stimulants, have been able to improve antibody and cellular responses in mice, but often at the expense of increased adverse effects. Therefore, extensive research has been devoted to the development of new adjuvants and new antigenic targets to minimize side-effects and increase the efficacy and immunogenicity of peptide-based malaria vaccines. In developing malaria vaccines and identifying new *Plasmodium* antigens, several methods are used, such as array technology, immunoinformatics, PCR deep sequencing technology and whole-proteome screening. Many antigens/proteins are yet to be utilized for vaccine development; screening for their antigenicity should be performed.

## Figures and Tables

**Table 1:** Pre-erythrocytic peptide-based vaccine constructs against malaria.

Vaccine Name	Parasite	Source of Peptide Antigens	Adjuvant	Immune Response	Clinical/Animal Trials	References
**RTS,S (GlaxoSmithKline Biologicals)**	*P. falciparum*	CSP (207–395); S (the N-terminus of HBsAg)	AS01, AS02	Protective humoral/cellular	Children and infants	[30,47,48]
**QNT-5**	*P. falciparum*	CD4 T-cell epitope from C-terminus of the CS protein	Montanide ISA 720	Protective humoral	HLA-DR4 transgenic mice	[49]
Tri-epitope CS peptide T1BT*	*P. falciparum*	B-cell epitope from CS repeat region and two T-cell epitopes: conserved T1 epitope and the universal epitope T*	Freund’s adjuvant	Protective cellular	C57BL/6J (H-2^b^) and BALB/cJ (H-2^d^) mice	[50]
**CS repeat peptide**	*P. falciparum*	A protective B-cell peptide epitope of CS repeat region	TLR agonists	Protective humoral	C57BL/6 mice	[51]
**NMRC-M3V-Ad-PfCA**	*P. falciparum*	CS protein; apical membrane antigen-1 (AMA-1)	ND	Protective cellular	Human adults	[52]
**Ad-CA, Ad-C**	*P. falciparum*	CS protein; AMA-1		Protective cellular	Human adults	[52]
**AMA49-L1 and AMA49-C1**	*P. falciparum*	Cyclic and linear AMA1(446–490) peptide	ND	Protective humoral	BALB/c mice	[53]
**UK-39 and AMA49-C1**	*P. falciparum*	UK-39 from CSP; AMA49-C1 peptides from AMA-1	ND	Protective humoral	Human adults	[54]
**AMA-1 and CSP**	*P. falciparum*	SCP; AMA-1	ND	Protective cellular	Human adults	[55]
**LSA-1**	*P. falciparum*	LSA-1	ND	No immune response	BALB/c mice	[56]
**LSA-1**	*P. falciparum*	LSA-1	ND	Protective humoral	Human adults	[57]
**LSA-3**	*P. falciparum*	LSA-3	ND	Protective cellular	Chimpanzees	[58,59]
**VMP001,**	*P. vivax*	N- and C- terminal regions of the CSV and a truncated repeat region that contains repeat sequences from both the VK210 (type 1) and the VK247 (type 2) parasites	AS01	Protective humoral/cellular	Rhesus monkeys	[60]
**CSV-S,S**	*P. vivax*	VMP001 and S (the N-terminus of HBsAg)	AS01	Protective humoral/cellular	Rhesus monkeys	[60]
**KI**	*P. berghei*	pb9 (SYIPSAEKI, referred to as KI) from CS protein	Montanide, Poly I:C	Protective humoral/cellular	BALB/c mice	[61]
**ACT-CS**	*P. berghei*	recombinant *Bordetella* adenylate cyclase toxoid fused with an MHC class I-restricted epitope of the CS protein	ND	Protective humoral/cellular	DEREG-mice	[62]

ND: no data. LSA-1: DNA sequence coding for a *P. falciparum* liver-stage-specific antigen composed of repeats of 17 amino-acids.

**Table 2:** Erythrocytic peptide-based vaccines constructs against malaria.

Vaccine Name	Parasite	Source of Peptide Antigen	Adjuvant	Immune Response	Clinical Trials/Animals	References
**MSP-1, MSP-3b, and GLURP**	*P. falciparum*	MSP; GLURP_27–500_; GLURP_489–705_; GLURP_705–1178_	ND	Protective humoral	Human adults	[82]
**MSP-3, GLURP, and AMA1**	*P. falciparum*	MSP-3_181–276_; GLURP_94–489_; R2 (repeat region amino acids 705–1178	ND	Protective humoral	Human adults	[83]
**MSP-3, GLURP**	*P. falciparum*	MSP_181–276_; GLURP_27–500_	ND	Recognized by natural immune response	Human adults	[84]
**GMZ2**	*P. falciparum*	Fusion of GLURP_27–500_ and MSP3_212–380_	Al(OH)_3_	Recognized by natural immune response	Human adults	[85]
**Spf66**	*P. falciparum*	Three merozoite-derived antigens and NANP epitope of CSP	ND	No/little efficacy	Human adults	[78,86,87]

MSP-3b: synthetic peptide (184-AKEASSYDYILGWEFGGGVPEHKKEEN-210); ND: no data.

**Table 3:** Recent clinical trials on vaccines against *P. falciparum.*

Vaccine Name	Target Protein	Vaccination Protocol	Clinical Phase	Antigen Source
***Pre-erythrocytic stage***				
**RTS,S/AS01E**	Pf CSP (207–395) and HepBsAg	IM	IV	*S. cerevisiae*
**ChAd63/MVA ME-TRAP**	TRAP + ME epitopes (CS, LSA1, LSA3, STARP, EXP1, pb9)	IM	IIb	Simian adenovirus ChAd63, MVA
**ChAd63/MVA ME-TRAP + Matrix M™**	TRAP + ME epitopes (CS, LSA1, LSA3, STARP, EXP1, pb9)	IM	I	Simian adenovirus ChAd63, MVA
**PfSPZ**	ND	DVI	ND	Simian adenovirus ChAd63, MVA
**PfCelTOS FMP012**	CelTOS (cell-traversal protein for ookinetes and sporozoites)	IM	Ia	*E. coli*, B834
**CSVAC**	CS	IM	I	Adenovirus ChAd63 and MVA
**R21/AS01B**	CSP	ND	Ia	ND
**R21/Matrix-M1**	CSP	ND	Ib	ND
**R21 (RTS,S-biosimilar)/ME-TRAP**	CSP	SC, ID, IM	IIa	*P. pastoris*
***Blood stage***				
**GMZ2**	GLURP, MSP3	IM	II	*L. lactis*
**PfAMA1-DiCo**	AMA-1-DiCo	IM	I	*P. pastoris*
**P27A**	P27A	IM	ND	Synthetic peptide
**MSP3[181–276]**	MSP3	SC	IIb	Synthetic peptide
**SE36**	N-terminal of serine repeat antigen (SERA5)	SC, IM	Ib	*E. coli*
**PfPEBS**	ND	ND	II	*E. coli*
**ChAd63 RH5 +/− MVA RH5**	RH	IM	Ia	ChAd63 and MVA
**PRIMVAC**	VAR2CSA	IM	Ia/b	*E. coli*
**PAMVAC**	VAR2CSA	IM	Ia/b	*Drosophila* S2 cells
***Sexual stage***				
**Pfs25 VLP**	Pfs25	IM	I/IIa	*N. benthamiana*
**Pfs25-EPA/Alhydrogel**	Pfs25	IM	Ib	*P. pastoris*
**Pfs230D1M-EPA/Alhydrogel and/or Pfs25-EPA/Alhydrogel**	Pfs25M, Pfs230D1M	IM	I	*P. pastoris*
**Pfs230D1M-EPA/Alhydrogel and Pfs25-EPA/AS01**	Pfs25M, Pfs230D1M	IM	I	*P. pastoris*
**ChAd63 Pfs25-IMX313/MVA Pfs25-IMX313**	Pfs25	IM	Ia	Chimpanzee Adenovirus 63, MVA

Data were collected from https://clinicaltrials.gov/; ND: no data; IM: intramuscular injection; SC: subcutaneous injection; DVI: direct venous inoculation; ID: intradermal injection.

## References

[1] Hay S.I., Omumbo J.A., Craig M.H., Snow R.W. (2000). Earth observation, geographic information systems and *Plasmodium falciparum* malaria in sub-Saharan Africa. Adv. Parasit..

[2] Humphreys M. (2001). Malaria: Poverty, race, and public health in the United States.

[3] Singh B., Sung L.K., Matusop A., Radhakrishnan A., Shamsul S.S.G., Cox-Singh J., Thomas A., Conway D.J. (2004). A large focus of naturally acquired *Plasmodium knowlesi* infections in human beings. Lancet.

[4] Guerra C.A., Gikandi P.W., Tatem A.J., Noor A.M., Smith D.L., Hay S.I., Snow R.W. (2008). The limits and intensity of *Plasmodium falciparum* transmission: Implications for malaria control and elimination worldwide. PloS Med..

[5] Guerra C.A., Howes R.E., Patil A.P., Gething P.W., Van Boeckel T.P., Temperley W.H., Kabaria C.W., Tatem A.J., Manh B.H., Elyazar I.R.F. (2010). The international limits and population at risk of *Plasmodium vivax* transmission in 2009. PloS Negl. Trop D.

[6] Gallup J.L., Sachs J.D. (2001). The economic burden of malaria. Am. J. Trop Med. Hyg..

[7] Menendez C. (1995). Malaria during pregnancy-a priority area of malaria research and control. Parasitol. Today.

[8] Monasch R., Reinisch A., Steketee R.W., Korenromp E.L., Alnwick D., Bergevin Y. (2004). Child coverage with mosquito nets and malaria treatment from population-based surveys in African countries: A baseline for monitoring progress in roll back malaria. Am. J. Trop Med. Hyg..

[9] Najera J.A., Gonzalez-Silva M., Alonso P.L. (2011). Some lessons for the future from the global malaria eradication programme (1955-1969). PloS Med..

[10] van den Berg H. (2009). Global status of DDT and its alternatives for use in vector control to prevent disease. Environ. Health Persp..

[11] Mabaso M.L.H., Sharp B., Lengeler C. (2004). Historical review of malarial control in southern African with emphasis on the use of indoor residual house-spraying. Trop. Med. Int. Health.

[12] Tren R., Bate R., Liberty Institute (New Delhi India) (2000). When Politics Kills: Malaria and the DDT Story.

[13] Peters W. (1990). *Plasmodium*: Resistance to antimalarial drugs. Ann. Parasitol. Hum. Comp..

[14] Wang J.G., Xu C.C., Wong Y.K., Li Y.J., Liao F.L., Jiang T.L., Tu Y.Y. (2019). Artemisinin, the magic drug discovered from traditional Chinese medicine. Engineering.

[15] Dahl E.L., Rosenthal P.J. (2007). Multiple antibiotics exert delayed effects against the *Plasmodium falciparum* anicoplast. Antimicrob. Agents Chemother..

[16] Gaillard T., Madamet M., Tsombeng F.F., Dormoi J., Pradines B. (2016). Antibiotics in malaria therapy: Which antibiotics except tetracyclines and macrolides may be used against malaria?. Malar. J..

[17] Wynands R., Affolderbach C., Hollberg L., Kitching J., Knappe S., Stahler M. (2002). Miniaturized laser magnetometers and clocks. Icono 2001 Quantum At. Opt. High Precis. Meas. Opt. Opt. Inf. Process. Transm. Storage.

[18] Perlmann P., Troye-Blomberg M. (2002). Malaria and the immune system in humans. Malar. Immunol..

[19] Greenwood B., Targett G. (2011). The mysteries of immunity to malaria. Lancet.

[20] Doolan D.L., Dobano C., Baird J.K. (2009). Acquired immunity to malaria. Clin. Microbiol. Rev..

[21] Ramharter M., Winkler H., Kremsner P.G., Adegnika A.A., Willheim M., Winkler S. (2005). Age-dependency of *Plasmodium falciparum*-specific and non-specific T cell cytokine responses in individuals from a malaria-endemic area. Eur. Cytokine Netw..

[22] Nussenzweig R.S., Vanderberg J., Most H., Orton C. (1967). Protective immunity produced by the injection of x-irradiated sporozoites of *Plasmodium berghei*. Nature.

[23] Giddam A.K., Reiman J.M., Zaman M., Skwarczynski M., Toth I., Good M.F. (2016). A semi-synthetic whole parasite vaccine designed to protect against blood stage malaria. Acta Biomater..

[24] Clyde D.F., Mccarthy V.C., Miller R.M., Woodward W.E. (1975). Immunization of man against *falciparum* and *vivax* malaria by use of attenuated sporozoites. Am. J. Trop Med. Hyg..

[25] Hoffman S.L., Goh L.M.L., Luke T.C., Schneider I., Le T.P., Doolan D.L., Sacci J., de la Vega P., Dowler M., Paul C. (2002). Protection of humans against malaria by immunization with radiation-attenuated *Plasmodium falciparum* sporozoites. J. Infect. Dis..

[26] Florens L., Washburn M.P., Raine J.D., Anthony R.M., Grainger M., Haynes J.D., Moch J.K., Muster N., Sacci J.B., Tabb D.L. (2002). A proteomic view of the *Plasmodium falciparum* life cycle. Nature.

[27] Bray R.S., Garnham P.C.C. (1982). The life-cycle of primate malaria parasites. Brit. Med. Bull..

[28] Huff C.G. (1947). Life cycle of malarial parasites. Annu. Rev. Microbiol..

[29] Gosling R., von Seidlein L. (2016). The future of the RTS,S/AS01 malaria vaccine: An alternative development plan. PloS Med..

[30] Tinto H., D’Alessandro U., Sorgho H., Valea I., Tahita M.C., Kabore W., Kiemde F., Lompo P., Ouedraogo S., Derra K. (2015). Efficacy and safety of RTS,S/AS01 malaria vaccine with or without a booster dose in infants and children in Africa: Final results of a phase 3, individually randomised, controlled trial. Lancet.

[31] Regules J.A., Cicatelli S.B., Bennett J.W., Paolino K.M., Twomey P.S., Moon J.E., Kathcart A.K., Hauns K.D., Komisar J.L., Qabar A.N. (2016). Fractional third and fourth dose of RTS,S/AS01 malaria candidate vaccine: A phase 2a controlled human malaria parasite infection and immunogenicity study. J. Infect. Dis..

[32] Nielsen C.M., Vekemans J., Lievens M., Kester K.E., Regules J.A., Ockenhouse C.F. (2018). RTS,S malaria vaccine efficacy and immunogenicity during *Plasmodium falciparum* challenge is associated with HLA genotype. Vaccine.

[33] Mackinnon M.J., Marsh K. (2010). The selection landscape of malaria parasites. Science.

[34] Bertolino P., Bowen D.G. (2015). Malaria and the liver: Immunological hide-and-seek or subversion of immunity from within?. Front. Microbiol..

[35] Hill A.V. (2011). Vaccines against malaria. Philos. Trans. R. Soc. Lond. B Biol. Sci..

[36] Draper S.J., Angov E., Horii T., Miller L.H., Srinivasan P., Theisen M., Biswas S. (2015). Recent advances in recombinant protein-based malaria vaccines. Vaccine.

[37] Skwarczynski M., Toth I. (2016). Peptide-based synthetic vaccines. Chem. Sci..

[38] Malonis R.J., Lai J.R., Vergnolle O. (2020). Peptide-based vaccines: Current progress and future challenges. Chem. Rev..

[39] Herrington D.A., Clyde D.F., Losonsky G., Cortesia M., Murphy J.R., Davis J., Baqar S., Felix A.M., Heimer E.P., Gillessen D. (1987). Safety and immunogenicity in man of a synthetic peptide malaria vaccine against *Plasmodium-falciparum* sporozoites. Nature.

[40] Gerberding J.L. (2013). The new vaccine frontier. SD Med..

[41] Beeson J.G., Drew D.R., Boyle M.J., Feng G., Fowkes F.J.I., Richards J.S. (2016). Merozoite surface proteins in red blood cell invasion, immunity and vaccines against malaria. Fems Microbiol. Rev..

[42] Gruner A.C., Snounou G., Brahimi K., Letourneur F., Renia L., Druilhe P. (2003). Pre-erythrocytic antigens of *Plasmodium falciparum*: From rags to riches?. Trends Parasitol..

[43] Richards J.S., Beeson J.G. (2009). The future for blood-stage vaccines against malaria. Immunol. Cell Biol..

[44] Bolad A., Berzins K. (2000). Antigenic diversity of *Plasmodium falciparum* and antibody-mediated parasite neutralization. Scand. J. Immunol..

[45] Kengne-Ouafo J.A., Sutherland C.J., Binka F.N., Awandare G.A., Urban B.C., Dinko B. (2019). Immune responses to the sexual stages of *plasmodium falciparum* parasites. Front. Immunol..

[46] Tachibana M., Suwanabun N., Kaneko O., Iriko H., Otsuki H., Sattabongkot J., Kaneko A., Herrera S., Torii M., Tsuboi T. (2015). *Plasmodium vivax* gametocyte proteins, Pvs48/45 and Pvs47, induce transmission-reducing antibodies by DNA immunization. Vaccine.

[47] White M.T., Bejon P., Olotu A., Griffin J.T., Riley E.M., Kester K.E., Ockenhouse C.F., Ghani A.C. (2013). The relationship between RTS,S vaccine-induced antibodies, CD4(+) T cell responses and protection against *Plasmodium falciparum* infection. PLoS ONE.

[48] Garcon N., Heppner D.G., Cohen J. (2003). Development of RTS,S/AS02: A purified subunit-based malaria vaccine candidate formulated with a novel adjuvant. Expert Rev. Vaccines.

[49] Parra-Lopez C.A., Bernal-Estevez D., Eduardo Vargas L., Pulido-Calixto C., Salazar L.M., Calvo-Calle J.M., Stern L.J. (2014). An unstable Th epitope of P. falciparum fosters central memory T cells and anti-CS antibody responses. PLoS ONE.

[50] Powell T.J., Tang J., DeRome M.E., Mitchell R.A., Jacobs A., Deng Y., Palath N., Cardenas E., Boyd J.G., Nardin E. (2013). *Plasmodium falciparum* synthetic LbL microparticle vaccine elicits protective neutralizing antibody and parasite-specific cellular immune responses. Vaccine.

[51] Mitchell R.A., Altszuler R., Frevert U., Nardin E.H. (2016). Skin scarification with *Plasmodium falciparum* peptide vaccine using synthetic TLR agonists as adjuvants elicits malaria sporozoite neutralizing immunity. Sci. Rep..

[52] Schwenk R., Banania G., Epstein J., Kim Y., Peters B., Belmonte M., Ganeshan H., Huang J., Reyes S., Stryhn A. (2013). Ex vivo tetramer staining and cell surface phenotyping for early activation markers CD38 and HLA-DR to enumerate and characterize malaria antigen-specific CD8(+) T-cells induced in human volunteers immunized with a *Plasmodium falciparum* adenovirus-vectored malaria vaccine expressing AMA1. Malar. J..

[53] Mueller M.S., Renard A., Boato F., Vogel D., Naegeli M., Zurbriggen R., Robinson J.A., Pluschke G. (2003). Induction of parasite growth-inhibitory antibodies by a virosomal formulation of a peptidomimetic of loop I from domain III of *Plasmodium falciparum* apical membrane antigen 1. Infect. Immun..

[54] Okitsu S.L., Silvie O., Westerfeld N., Curcic M., Kammer A.R., Mueller M.S., Sauerwein R.W., Robinson J.A., Genton B., Mazier D. (2007). A virosomal malaria peptide vaccine elicits a long-lasting sporozoite-inhibitory antibody response in a phase 1a clinical trial. PLoS ONE.

[55] Cech P.G., Aebi T., Abdallah M.S., Mpina M., Machunda E.B., Westerfeld N., Stoffel S.A., Zurbriggen R., Pluschke G., Tanner M. (2011). Virosome-formulated *Plasmodium falciparum* AMA-1 & CSP derived peptides as malaria vaccine: Randomized phase 1b trial in semi-immune adults & children. PLoS ONE.

[56] Guerinmarchand C., Druilhe P., Galey B., Londono A., Patarapotikul J., Beaudoin R.L., Dubeaux C., Tartar A., Mercereaupuijalon O., Langsley G. (1987). A liver-stage-specific antigen of *Plasmodium-falciparum* characterized by gene cloning. Nature.

[57] Pratt-Riccio L.R., De Souza Perce-Da-Silva D., Da Costa Lima-Junior J., Pratt Riccio E.K., Ribeiro-Alves M., Santos F., Arruda M., Camus D., Druilhe P., Oliveira-Ferreira J. (2017). Synthetic antigens derived from *Plasmodium falciparum* sporozoite, liver, and blood stages: Naturally acquired immune response and human leukocyte antigen associations in individuals living in a Brazilian endemic area. Am. J. Trop Med. Hyg..

[58] Daubersies P., Thomas A.W., Millet P., Brahimi K., Langermans J.A.M., Ollomo B., Ben Mohamed L., Slierendregt B., Eling W., Van Belkum A. (2000). Protection against *Plasmodium falciparum* malaria in chimpanzees by immunization with the conserved preerythrocytic liver-stage antigen 3. Nat. Med..

[59] Daubersies P., Ollomo B., Sauzet J.P., Brahimi K., Perlaza B.L., Eling W., Moukana H., Rouquet P., de Taisne C., Druilhe P. (2008). Genetic immunisation by liver stage antigen 3 protects chimpanzees against malaria despite low immune responses. PLoS ONE.

[60] Vanloubbeeck Y., Pichyangkul S., Bayat B., Yongvanitchit K., Bennett J.W., Sattabongkot J., Schaecher K., Ockenhouse C.F., Cohen J., Yadava A. (2013). Comparison of the immune responses induced by soluble and particulate *Plasmodium vivax* circumsporozoite vaccine candidates formulated in AS01 in rhesus macaques. Vaccine.

[61] Wilson K.L., Xiang S.D., Plebanski M. (2016). A model to study the impact of polymorphism driven liver-stage immune evasion by malaria parasites, to help design effective cross-reactive vaccines. Front. Microbiol..

[62] Mora M.d.R.E., Steeg C., Tartz S., Heussler V., Sparwasser T., Link A., Fleischer B., Jacobs T. (2014). Depletion of regulatory T cells augments a vaccine-induced T effector cell response against the liver-stage of malaria but fails to increase memory. PLoS ONE.

[63] Kumar K.A., Sano G., Boscardin S., Nussenzweig R.S., Nussenzweig M.C., Zavala F., Nussenzweig V. (2006). The circumsporozoite protein is an immunodominant protective antigen in irradiated sporozoites. Nature.

[64] Zou X.Y., House B.L., Zyzak M.D., Richie T.L., Gerbasi V.R. (2013). Towards an optimized inhibition of liver stage development assay (ILSDA) for *Plasmodium falciparum*. Malar. J..

[65] Gordon D.M., Mcgovern T.W., Krzych U., Cohen J.C., Schneider I., Lachance R., Heppner D.G., Yuan G., Hollingdale M., Slaoui M. (1995). Safety, immunogenicity, and efficacy of a recombinantly produced *Plasmodium-falciparum* circumsporozoite-protein hepatitis-B surface-antigen subunit vaccine. J. Infect. Dis..

[66] Cohen J.D., Cohen J. (2007). Use of *Plasmodium* antigen and adjuvant having lipid A derivative and saponin, in manufacture of medicament or in manufacture of kit, for immunizing travelers to endemic regions against productive malaria infection.

[67] CalvoCalle J.M., Hammer J., Sinigaglia F., Clavijo P., MoyaCastro Z.R., Nardin E.H. (1997). Binding of malaria T cell epitopes to DR and DQ molecules in vitro correlates with immunogenicity in vivo-Identification of a universal T cell epitope in the *Plasmodium falciparum* circumsporozoite protein. J. Immunol..

[68] Deoliveira G.A., Clavijo P., Nussenzweig R.S., Nardin E.H. (1994). Immunogenicity of an alum-adsorbed synthetic multiple-antigen peptide-based on B-cell and T-cell epitopes of the *Plasmodium-falciparum* CS-protein-possible vaccine application. Vaccine.

[69] Nardin E. (2010). The past decade in malaria synthetic peptide vaccine clinical trials. Hum. Vaccin..

[70] Wilson K.L., Xiang S.D., Plebanski M. (2015). Montanide, Poly I:C and nanoparticle based vaccines promote differential suppressor and effect or cell expansion: A study of induction of CD8 T cells to a minimal *Plasmodium berghei* epitope. Front. Microbiol..

[71] Chandrudu S., Skwarczynski M., Pattinson D., Apte S.H., Doolan D.L., Toth I. (2016). Synthesis and immunological evaluation of peptide-based vaccine candidates against malaria. Biochem. Compd..

[72] Apte S.H., Groves P.L., Skwarczynski M., Fujita Y., Chang C.H., Toth I., Doolan D.L. (2012). Vaccination with lipid core peptides fails to induce epitope-specific T cell responses but confers non-specific protective immunity in a malaria model. PLoS ONE.

[73] Schwartz L., Brown G.V., Genton B., Moorthy V.S. (2012). A review of malaria vaccine clinical projects based on the WHO rainbow table. Malar. J..

[74] Fidock D.A., Grasmasse H., Lepers J.P., Brahimi K., Benmohamed L., Mellouk S., Guerinmarchand C., Londono A., Raharimalala L., Meis J.F.G.M. (1994). *Plasmodium-falciparum* liver stage antigen-1 is well conserved and contains potent B-cell and T-cell determinants. J. Immunol..

[75] Mikolajczak S.A., Sacci J.B., De La Vega P., Camargo N., VanBuskirk K., Krzych U., Cao J., Jacobs-Lorena M., Cowman A.F., Kappe S.H. (2011). Disruption of the *Plasmodium falciparum* liver-stage antigen-1 locus causes a differentiation defect in late liver-stage parasites. Cell Microbiol..

[76] Druilhe P., Guerinmarc C., Guerinmarchand C. (1990). New poly peptide used to diagnose malaria-contg. sequences from protein of sporozoite stage of plasmodium falciparum.

[77] Cummings J.F., Spring M.D., Schwenk R.J., Ockenhouse C.F., Kester K.E., Polhemus M.E., Walsh D.S., Yoon I.K., Prosperi C., Juompan L.Y. (2010). Recombinant Liver Stage Antigen-1 (LSA-1) formulated with AS01 or AS02 is safe, elicits high titer antibody and induces IFN-gamma/IL-2 CD4+ T cells but does not protect against experimental *Plasmodium falciparum* infection. Vaccine.

[78] (2016). The challenges of introducing a malaria vaccine. Bull World Health Organ.

[79] Goh Y.S., McGuire D., Renia L. (2019). Vaccination with Sporozoites: Models and Correlates of Protection. Front. Immunol..

[80] Mak T.W., Saunders M.E., Mak T.W., Saunders M.E. (2006). 23-Vaccines and Clinical Immunization. The Immune Response.

[81] Ouattara A., Laurens M.B. (2014). Vaccines against malaria. Clin. Infect. Dis..

[82] Soe S., Theisen M., Roussilhon C., Aye K.S., Druilhe P. (2004). Association between protection against clinical malaria and antibodies to merozoite surface antigens in an area of hyperendemicity in Myanmar: Complementarity between responses to merozoite surface protein 3 and the 220-kilodalton glutamate-rich protein. Infect. Immun..

[83] Nebie I., Tiono A.B., Diallo D.A., Samandoulougou S., Diarra A., Konate A.T., Cuzin-Ouattara N., Theisen M., Corradin G., Cousens S. (2008). Do antibody responses to malaria vaccine candidates influenced by the level of malaria transmission protect from malaria?. Trop Med. Int. Health.

[84] Amoah L.E., Nuvor S.V., Obboh E.K., Acquah F.K., Asare K., Singh S.K., Boampong J.N., Theisen M., Williamson K.C. (2017). Natural antibody responses to *Plasmodium falciparum* MSP3 and GLURP(R0) antigens are associated with low parasite densities in malaria patients living in the Central Region of Ghana. Parasites Vectors.

[85] Jepsen M.P., Jogdand P.S., Singh S.K., Esen M., Christiansen M., Issifou S., Hounkpatin A.B., Ateba-Ngoa U., Kremsner P.G., Dziegiel M.H. (2013). The malaria vaccine candidate GMZ2 elicits functional antibodies in individuals from malaria endemic and non-endemic areas. J. Infect. Dis..

[86] Graves P., Gelband H. (2006). Vaccines for preventing malaria (SPf66). Cochrane Database Syst. Rev..

[87] (2016). U.S. medical eligibility criteria for contraceptive use, 2016. Centers for Disease Control and Prevention-Morbidity and Mortality Weekly Report.

[88] Holder A.A. (2009). The carboxy-terminus of merozoite surface protein 1: Structure, specific antibodies and immunity to malaria. Parasitology.

[89] Chatterjee S., Sharma P., Kumar S., Chauhan V.S. (1995). Fine specificity of immune responses to epitopic sequences in synthetic peptides containing B and T epitopes from the conserved *Plasmodium falciparum* blood-stage antigens. Vaccine.

[90] Lougovskoi A.A., Okoyeh N.J., Chauhan V.S. (1999). Mice immunised with synthetic peptide from N-terminal conserved region of merozoite surface antigen-2 of human malaria parasite Plasmodium falciparum can control infection induced by *Plasmodium yoelii yoelii* 265BY strain. Vaccine.

[91] Sirima S.B., Mordmuller B., Milligan P., Ngoa U.A., Kironde F., Atuguba F., Tiono A.B., Issifou S., Kaddumukasa M., Bangre O. (2016). A phase 2b randomized, controlled trial of the efficacy of the GMZ2 malaria vaccine in African children. Vaccine.

[92] Theisen M., Adu B., Mordmuller B., Singh S. (2017). The GMZ2 malaria vaccine: From concept to efficacy in humans. Expert Rev. Vaccines.

[93] Wilson K.L., Flanagan K.L., Prakash M.D., Plebanski M. (2019). Malaria vaccines in the eradication era: Current status and future perspectives. Expert Rev. Vaccines.

[94] Amador R., Moreno A., Murillo L.A., Sierra O., Saavedra D., Rojas M., Mora A.L., Rocha C.L., Alvarado F., Falla J.C. (1992). Safety and immunogenicity of the synthetic malaria vaccine Spf66 in a large field trial. J. Infect. Dis..

[95] Salcedo M., Barreto L., Rojas M., Moya R., Cote J., Patarroyo M.E. (1991). Studies on the humoral immune-response to a synthetic vaccine against *Plasmodium-falciparum* malaria. Clin. Exp. Immunol..

[96] Valero M.V., Amador L.R., Galindo C., Figueroa J., Bello M.S., Murillo L.A., Mora A.L., Patarroyo G., Rocha C.L., Rojas M. (1993). Vaccination with Spf66, a chemically synthesized vaccine, against *Plasmodium-falciparum* malaria in Colombia. Lancet.

[97] Alonso P.L., Tanner M., Smith T., Hayes R.J., Schellenberg J.A., Lopez M.C., Deazevedo I.B., Menendez C., Lyimo E., Weiss N. (1994). A trial of the synthetic malaria vaccine Spf66 in Tanzania-rationale and design. Vaccine.

[98] Dalessandro U., Leach A., Drakeley C.J., Bennett S., Olaleye B.O., Fegan G.W., Jawara M., Langerock P., George M.O., Targett G.A.T. (1995). Efficacy trial of malaria vaccine Spf66 in Gambian infants. Lancet.

[99] Nosten F., Luxemburger C., Kyle D.E., Ballou W.R., Wittes J., Wah E., Chongsuphajaisiddhi T., Gordon D.M., White N.J., Sadoff J.C. (1996). Randomised double-blind placebo-controlled trial of SPf66 malaria vaccine in children in northwestern Thailand. Lancet.

[100] Horii T., Shirai H., Jie L., Ishii K.J., Palacpac N.Q., Tougan T., Hato M., Ohta N., Bobogare A., Arakaki N. (2010). Evidences of protection against blood-stage infection of *Plasmodium falciparum* by the novel protein vaccine SE36. Parasitol. Int..

[101] Pang X.L., Mitamura T., Horii T. (1999). Antibodies reactive with the N-terminal domain of *Plasmodium falciparum* serine repeat antigen inhibit cell proliferation by agglutinating merozoites and schizonts. Infect. Immun..

[102] Palacpac N.M.Q., Ntege E., Yeka A., Balikagala B., Suzuki N., Shirai H., Yagi M., Ito K., Fukushima W., Hirota Y. (2013). Phase 1b randomized trial and follow-up study in Uganda of the blood-stage malaria vaccine candidate BK-SE36. PLoS ONE.

[103] Venkatraman N., Anagnostou N., Bliss C., Bowyer G., Wright D., Lovgren-Bengtsson K., Roberts R., Poulton I., Lawrie A., Ewer K. (2017). Safety and immunogenicity of heterologous prime-boost immunization with viral-vectored malaria vaccines adjuvanted with Matrix-M (TM). Vaccine.

[104] Cox R.J., Pedersen G., Madhun A.S., Svindland S., Saevik M., Breakwell L., Hoschler K., Willemsen M., Campitelli L., Nostbakken J.K. (2011). Evaluation of a virosomal H5N1 vaccine formulated with Matrix M (TM) adjuvant in a phase I clinical trial. Vaccine.

[105] Bigaeva E., van Doorn E., Liu H., Hak E. (2016). Meta-Analysis on Randomized Controlled Trials of Vaccines with QS-21 or ISCOMATRIX Adjuvant: Safety and Tolerability. PLoS ONE.

[106] Bengtsson K.L., Karlsson K.H., Magnusson S.E., Reimer J.M., Stertman L. (2013). Matrix-M (TM) adjuvant: Enhancing immune responses by ‘setting the stage’ for the antigen. Expert Rev. Vaccines.

[107] Bengtsson K.L., Song H., Stertman L., Liu Y., Flyer D.C., Massare M.J., Xu R.H., Zhou B., Lu H., Kwilas S.A. (2016). Matrix-M adjuvant enhances antibody, cellular and protective immune responses of a Zaire Ebola/Makona virus glycoprotein (GP) nanoparticle vaccine in mice. Vaccine.

[108] McCall M.B.B., Kremsner P.G., Mordmuller B. (2018). Correlating efficacy and immunogenicity in malaria vaccine trials. Semin. Immunol..

[109] Julien J.P., Wardemann H. (2019). Antibodies against Plasmodium falciparum malaria at the molecular level. Nat. Rev. Immunol..

[110] Lee W.C., Russell B., Sobota R.M., Ghaffar K., Howland S.W., Wong Z.X., Maier A.G., Dorin-Semblat D., Biswas S., Gamain B. (2020). Plasmodium-infected erythrocytes induce secretion of IGFBP7 to form type II rosettes and escape phagocytosis. Elife.

[111] Wang R.B., Smith J.D., Kappe S.H.I. (2009). Advances and challenges in malaria vaccine development. Expert Rev. Mol. Med..

[112] Ssemaganda A., Giddam A.K., Zaman M., Skwarczynski M., Toth I., Stanisic D.I., Good M.F. (2019). Induction of *Plasmodium*-specific immune responses using liposome-based vaccines. Front. Immunol..

[113] Vandoolaeghe P., Schuerman L. (2016). The RTS,S/AS01 malaria vaccine in children 5 to 17 months of age at first vaccination. Expert Rev. Vaccines.

[114] Jindal H., Bhatt B., Malik J.S., Sk S., Mehta B. (2014). Malaria vaccine: A step toward elimination. Hum. Vaccin. Immunother..

[115] Mitran C.J., Mena A., Gnidehou S., Banman S., Arango E., Lima B.A.S., Lugo H., Ganesan A., Salanti A., Mbonye A.K. (2019). Antibodies to cryptic epitopes in distant homologues underpin a mechanism of heterologous immunity between Plasmodium vivax PvDBP and Plasmodium falciparum VAR2CSA. MBio.

[116] Pandey R.K., Bhatt T.K., Prajapati V.K. (2018). Novel immunoinformatics approaches to design multi-epitope subunit vaccine for malaria by investigating *Anopheles* salivary protein. Sci. Rep..

[117] Karch C.P., Doll T., Paulillo S.M., Nebie I., Lanar D.E., Corradin G., Burkhard P. (2017). The use of a *P. falciparum* specific coiled-coil domain to construct a self-assembling protein nanoparticle vaccine to prevent malaria. J. Nanobiotechnol..

[118] Loeffler F.F., Pfeil J., Heiss K. (2016). High-density peptide arrays for malaria vaccine development. Methods Mol. Biol. (CliftonN.J.).

[119] Guleria V., Jaiswal V. (2019). Comparative transcriptome analysis of different stages of *Plasmodium falciparum* to explore vaccine and drug candidates. Genomics.

